# Strategies for generating mouse model resources of human disease

**DOI:** 10.1093/procel/pwad011

**Published:** 2023-03-14

**Authors:** Jirong Pan, Ling Zhang, Zhibing Huang, Dalu Zhao, He Li, Yanan Fu, Meng Wang, Borui Chen, Fuad A Iraqi, Grant Morahan, Chuan Qin

**Affiliations:** National Human Diseases Animal Model Resource Center, NHC Key Laboratory of Human Disease Comparative Medicine, Beijing Key Laboratory for Animal Models of Emerging and Reemerging Infectious Diseases, Beijing Engineering Research Center for Experimental Animal Models of Human Critical Diseases, Institute of Laboratory Animal Sciences, CAMS & PUMC, National Center of Technology Innovation for Animal Model, Changping National Laboratory (CPNL), Beijing 102206, China; National Human Diseases Animal Model Resource Center, NHC Key Laboratory of Human Disease Comparative Medicine, Beijing Key Laboratory for Animal Models of Emerging and Reemerging Infectious Diseases, Beijing Engineering Research Center for Experimental Animal Models of Human Critical Diseases, Institute of Laboratory Animal Sciences, CAMS & PUMC, National Center of Technology Innovation for Animal Model, Changping National Laboratory (CPNL), Beijing 102206, China; National Human Diseases Animal Model Resource Center, NHC Key Laboratory of Human Disease Comparative Medicine, Beijing Key Laboratory for Animal Models of Emerging and Reemerging Infectious Diseases, Beijing Engineering Research Center for Experimental Animal Models of Human Critical Diseases, Institute of Laboratory Animal Sciences, CAMS & PUMC, National Center of Technology Innovation for Animal Model, Changping National Laboratory (CPNL), Beijing 102206, China; National Human Diseases Animal Model Resource Center, NHC Key Laboratory of Human Disease Comparative Medicine, Beijing Key Laboratory for Animal Models of Emerging and Reemerging Infectious Diseases, Beijing Engineering Research Center for Experimental Animal Models of Human Critical Diseases, Institute of Laboratory Animal Sciences, CAMS & PUMC, National Center of Technology Innovation for Animal Model, Changping National Laboratory (CPNL), Beijing 102206, China; National Human Diseases Animal Model Resource Center, NHC Key Laboratory of Human Disease Comparative Medicine, Beijing Key Laboratory for Animal Models of Emerging and Reemerging Infectious Diseases, Beijing Engineering Research Center for Experimental Animal Models of Human Critical Diseases, Institute of Laboratory Animal Sciences, CAMS & PUMC, National Center of Technology Innovation for Animal Model, Changping National Laboratory (CPNL), Beijing 102206, China; National Human Diseases Animal Model Resource Center, NHC Key Laboratory of Human Disease Comparative Medicine, Beijing Key Laboratory for Animal Models of Emerging and Reemerging Infectious Diseases, Beijing Engineering Research Center for Experimental Animal Models of Human Critical Diseases, Institute of Laboratory Animal Sciences, CAMS & PUMC, National Center of Technology Innovation for Animal Model, Changping National Laboratory (CPNL), Beijing 102206, China; National Human Diseases Animal Model Resource Center, NHC Key Laboratory of Human Disease Comparative Medicine, Beijing Key Laboratory for Animal Models of Emerging and Reemerging Infectious Diseases, Beijing Engineering Research Center for Experimental Animal Models of Human Critical Diseases, Institute of Laboratory Animal Sciences, CAMS & PUMC, National Center of Technology Innovation for Animal Model, Changping National Laboratory (CPNL), Beijing 102206, China; National Human Diseases Animal Model Resource Center, NHC Key Laboratory of Human Disease Comparative Medicine, Beijing Key Laboratory for Animal Models of Emerging and Reemerging Infectious Diseases, Beijing Engineering Research Center for Experimental Animal Models of Human Critical Diseases, Institute of Laboratory Animal Sciences, CAMS & PUMC, National Center of Technology Innovation for Animal Model, Changping National Laboratory (CPNL), Beijing 102206, China; Department of Clinical Microbiology and Immunology, Sackler Faculty of Medicine, Tel-Aviv University, Ramat Aviv, Tel Aviv 69978, Israel; Harry Perkins Institute of Medical Research, QEII Medical Centre and Centre for Medical Research, University of Western Australia, Nedlands, Perth, WA 6009, Australia; National Human Diseases Animal Model Resource Center, NHC Key Laboratory of Human Disease Comparative Medicine, Beijing Key Laboratory for Animal Models of Emerging and Reemerging Infectious Diseases, Beijing Engineering Research Center for Experimental Animal Models of Human Critical Diseases, Institute of Laboratory Animal Sciences, CAMS & PUMC, National Center of Technology Innovation for Animal Model, Changping National Laboratory (CPNL), Beijing 102206, China

## Abstract

**Graphical Abstract:**
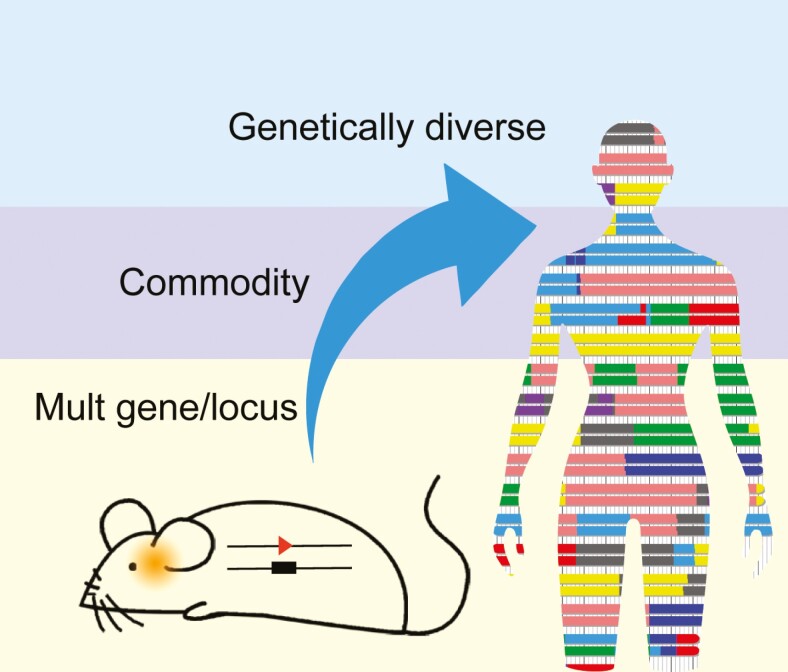

The number of genetically modified mouse models that mimic human disease is growing rapidly, but only a tiny fraction has been commonly used. According to The Knockout Mouse Program (Lloyd, 2011), a public resource of mouse embryonic stem cells containing a null mutation in every gene in the mouse genome, 8,916 mutant mice lines were phenotyped up to 19 July 2022. Due to the poor correlation between the genomic responses in the mouse models and those responses in human disease, and since humans differ significantly in their genetic vulnerability to common diseases, we still need better mouse models, especially for common and chronic human diseases, including cancer, pulmonary and cardiovascular diseases, obesity and diabetes, behavioral disorders, and neurodegenerative diseases. These new models will be placed into a public repository, The China National Human Disease Animal Model Resource Center (NAMR). This project is funded by Ministry of Science and Technology of China and specializes in the creation, introduction, collection, preservation, and supply of animal model resources for human diseases.

From a methodological perspective, two strategies are used to study the effects of genetic variations. “Forward genetics” is a phenotype-driven approach where naturally occurring or induced mutations are selected based on the phenotype they mediate; the variations determine the molecular causes for the morphological or physiological defects observed (Visscher et al., 2021). In contrast, “Reverse genetics” is a DNA-driven approach, that starts with the targeted disruption of a specific genetic element, then follows up on the study of the effects of this mutation on the phenotype of an organism (Fig. 1) (Su et al., 2019). Reverse genetic models include transgenic, gene knockout, and humanized mice. These are ideal for studying previously identified elements of the genome whose functions are unknown. This has been the dominant approach for mouse model development.

**Figure 1.**
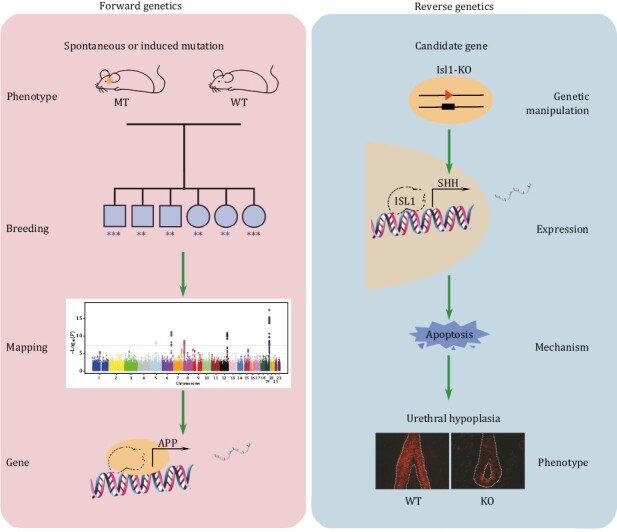
**Methodological view of animal models of human disease.** Forward genetics is a phenotype-driven approach, determining the genetic basis responsible for human disease, and reverse genetics is a DNA-driven approach. The cumulative effect of genes or mutations is an essential methodological basis for mouse model development.

However, most human diseases are not caused by single gene mutations but rather by the cumulative effect of mutations at several different loci interacting with significant environmental factors. Therefore, other strategies are required to generate more relevant animal models. We are going to generate a large number of animal models of human diseases using the following strategies: (i) multi-gene/loci mouse model for human diseases; (ii) comorbid mouse models for studying human comorbidity relationship; (iii) genetically diverse mouse models provide a rich resource for human disease models.

## Multi-gene/loci-modified mouse model for complex human diseases

Most human diseases result from dysfunction of elaborate cellular and molecular pathways involving multiple genes and/or complex gene expression patterns. Each of these genes [or as they are referred to when defined by genetic mapping analyses “quantitative trait loci” (QTL)] has only a weak effect, and it is only when an individual inherits several risk alleles at these QTLs that disease or disease predisposition ensues (Khera et al., 2018; Zhang et al., 2018).

The Alzheimer’s disease (AD) mouse model highlights this complexity, requiring breeding compound conditional lines of mice to replicate human disease, demonstrating the multi-gene/loci breeding process. AD is characterized by progressive cognitive impairment, usually beginning as memory loss but eventually progressing to involve multiple cognitive and behavioral domains. Pathologically, the disease is recognized by the presence of senile plaques, neurofibrillary tangles (NFTs), and neuronal loss (Braak and Braak, 1991; Hyman et al., 2012). The existence of familial mutations, such as APP695, APP751, APP770, the Swedish mutation (K670N/M671L), London (V717I), Indiana (V717F), Dutch (E693Q), and Arctic (E693G) is often cited as evidence that Aβ accumulation is central to AD pathogenesis (Selkoe and Hardy, 2016). Therefore, Amyloid precursor protein (App) isoforms and mutations were extensively chosen to be modified in the commonly used mouse models, but these APP transgenic mice only recapitulate some of the neuropathological features of human AD and other genes also need to be considered. *Presenilin* (PS1/2) mutant further reconstituted the remaining pathology, such as in APP/PS1 (Liu et al., 2017), 5×FAD (Oakley et al., 2006), and 3×Tg (Oddo et al., 2003) mouse model. The combination of PS1/2 mice with human APP‐overexpressing Tg mice increased pathogenic Aβ production which resulted in accelerated Aβ deposition and conferred amyloidogenicity, behavioral deficits, and neuronal loss (Oakley et al., 2006). In efforts to replicate NFT pathology, crossbreeding of mice that overexpress APP_swe_, and Tau_P301L_ transgenes on a PS1_M146V_ knock‐in background, enhanced tau pathology in the limbic system and olfactory cortex without affecting Aβ pathology (Oddo et al., 2003). More genes and loci with stronger genetic effects are still being discovered, and the permutation and combination of these lines will be bred to create better mouse models.

A panel of new AD model resources with immune system gene modification expands mouse models for studying the role of inflammation.

Inflammation is a central mechanism linking AD with other chronic diseases (Newman et al., 2005; Pugazhenthi et al., 2017). Several studies have suggested that neuroinflammation is fundamental in the pathogenesis of AD and contributes as much to pathology as do Aβ plaques and NFT (Pennisi et al., 2017). To establish a series of genetically modified mouse models related to inflammatory components, we searched relevant literature. We selected a panel of immune system genetic modified transgenic mice to breed mouse resources as a component of the NAMR. We intended to build a Repository of new AD model resources with deficiencies in inflammatory system components, including complement, cytokines, and chemokines.

**Figure 2.**
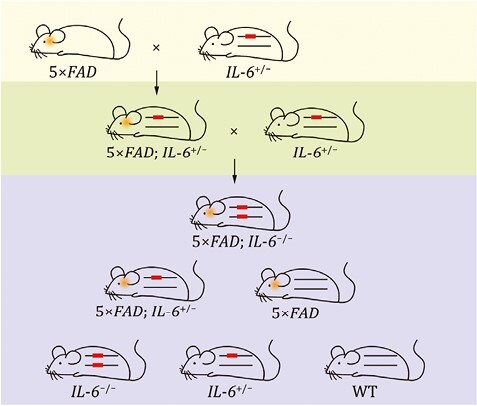
**The breeding process of AD model resources with immune system gene modification.** *IL-6* ^+/−^ mice are one of the immune system gene-modified mice panel, crossed with *5*×*FAD* mice, and then the resulting *5*×*FAD*; *IL-6*^+/−^ F1 mice were backcrossed with *IL-6*^+/−^ mice. *5*×*FAD*;*IL-6*^−/−^ mice were obtained and selected for experiments.

Our breeding strategy is consistent with previous reports in the literature (Fig. 2) (Cao et al., 2022), *IL-6*^+/−^ mice were crossed with *5*×*FAD* mice, and then the resulting *5*×*FAD*;*IL-6*^+/−^ F1 mice were backcrossed with *IL-6*^+/−^ mice. Among their progeny, *5*×*FAD*;*IL-6*^−/−^ mice were obtained, and tested at 3 and 10 months of age. Compared with *5*×*FAD* mice at the same age, 3-month-old *5*×*FAD*;*IL-6*^−/−^ mice had better Morris water maze space exploration performance, with significantly increased target quadrant residence time (*P* = 0.02) and the number of cross-platform times (*P* = 0.02). Immunohistochemical staining showed that these mice had reduced Aβ deposition in the brain. The results of phenotypic evaluation experiments showed that the mice with IL-6 gene knockout could significantly improve the spatial memory ability of 3-month-old *5×FAD* mice. These mice and phenotypic data represent a unique resource for scientists to identify immune system factors that influence resilience or vulnerability to AD.

## The modular design of human comorbidity mouse models

Modular design is a principle that subdivides a system into smaller parts called modules, which can be independently created, modified, replaced, or exchanged with other modules or between different systems. We propose that it is an effective method to cross disease modules (mice carrying different disease-related genes) to form comorbid mouse model resources for comorbidity research. The chances of obtaining a mouse model with relevance to human comorbidity are meager using either large-scale spontaneous mutation screening or gene editing techniques to generate transgenic mice (Stottmann and Beier, 2014; Gurumurthy et al., 2021). Humans have a high probability of having two or more diseases simultaneously. For instance, numerous studies reported that patients with diabetes have an increased risk of developing AD compared with healthy individuals (Biessels et al., 2010; Hirabayashi et al., 2016). Alzheimer transgenic mice (APP23) were crossed with two types of diabetic mice (ob/ob and NSY mice) and the metabolic and brain pathology of their offspring were analyzed. The onset of diabetes exacerbated Alzheimer-like cognitive dysfunction without an increase in brain amyloid-beta burden in double-mutant (APP(+)-ob/ob) mice. (Takeda et al., 2010). Also, AD Tg2576 mice were crossed with neuron-specific IR knockout mice (nIR^−/−^), showing that neuronal IR deficiency in Tg2576 (nIR^−/−^Tg2576) mice leads to markedly decreased Aβ burden (Stöhr et al., 2013). These results demonstrate that this approach is feasible for creating comorbidity models.

## Genetically diverse mice provide a powerful resource for human disease models

Controlled and standardized investigations of the genetics of complex human diseases are challenging because of the cumulative effect and interactions of numerous genes and environmental factors. Despite major discoveries of genetic risk factors for some diseases, the specific genes involved in susceptibility to most complex diseases and the mechanisms translating genetic effects into disease susceptibility are commonly unknown. There is a critical need, therefore, for new computational and experimental approaches dedicated to genetic analysis of complex human diseases.

It is often said that “there are no good mouse models” for a particular human disease. Most studies consider only a small number of closely related strains. Worse, nearly all the transgenic and KO mouse models produced have been made using a small number of mouse strains, usually C57BL/6. The host genetic background can have a significant effect on phenotype, and the limited number of strains available is a significant restriction imposed on generating mouse models. The collaborative cross (CC) mice may offer a great resource for solving this problem by capturing over 90% of the common genetic variation of the mouse species, and providing an extensive collection of isogenic strains that can be assessed under different environmental and other experimental conditions (Churchill et al., 2004). The genetic diversity of the CC has generated several animal models that faithfully replicate and mimic human diseases, including diabetic retinopathy (Weerasekera et al., 2015) and myelodysplastic syndrome (Li et al., 2021).

Furthermore, the diversity outbred (DO) mice and recombinant inbred inter-cross (RIX) strains can be adapted to induce genetic diversity to the currently used gene-modified mouse model to construct genetically diverse mouse model resources. The DO mice are a heterogeneous stock derived from 8 inbred founder strains, selected to maximize genetic diversity, and maintained by randomized breeding (Svenson et al., 2012). Each DO mouse is a unique individual with a high level of allelic heterozygosity. This heterogeneous stock provides an excellent opportunity to study medically relevant traits in a powerful model system and also gives investigators the power to complete high-resolution mapping of loci associated with disease phenotypes (Bufi and Korstanje, 2022). RI strains are inbred strains derived from the systematic inbreeding of randomly selected pairs of the F_2_ generation of a cross between two different inbred strains of mice (Threadgill et al., 2011). The CC and RIX strains can provide unlimited material for analysis.

In our unpublished data, APP_Swe_/PS1_DeltaE9_ mice were crossed with 21 CC strains to establish genetically diverse AD mouse models, for instance, the B6.WSB-APP_Swe_/PS1_DeltaE9_ F1 mice, and 6-month-old offspring mice were selected for experiments. Morris water maze space exploration results show that compared with C57BL6/J background mice at the same age, 6-month-old GD-AD mice target quadrant residence time and the number of cross-platform times varied significantly. These mice and phenotypic data represent a unique resource for scientists to identify unknown genes that influence resilience or vulnerability to AD. Some strains can be selected to be bred onto an inbred background for further research.

## Genetic information database and biobank of mouse models of human diseases

**Figure 3.**
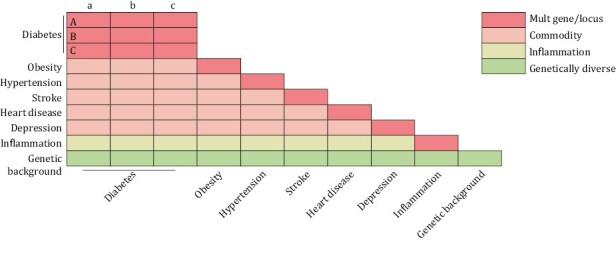
**Schematic representation of the breeding strategy for mouse models of human disease.** Using the cumulative effect of genes or locus mutations to construct multi-gene/locus-modified mouse models, using disease mouse modules to cross-breed comorbidity mouse models, and using genetically diverse mouse models to provide a genetic environment for human disease models. These approaches could generate thousands of mouse models of human disease.

Thousands of animal models are about to be created (Fig. 3), centralized repositories are essential, and we will build a mouse genetic information database and biobank of human diseases. There are many advantages of mice for constructing Database and Biobank of Human Diseases used for clinically relevant studies:

1. The number of human disease mouse models can be counted so that the sample size of the repositories is much smaller, making it financially affordable.
2. Most Gene-modified and multiple-gene/Luci mouse models can be maintained as homozygous. Since mouse models are reproducible, only several pairs of breeding mice need to be maintained (or stored as frozen embryos or frozen sperm) to provide mice in a shorter period instead of being bred from scratch.
3. RIX strains bred from genetically diverse mouse inbred strains. As long as the parent strains are alive, they can be crossed at any time to obtain mice with the same defined genome. With enormous sample sizes generated by high-throughput sequencing and other phenotypic data, followed by imputation, we will gain a very good understanding and complete picture of the gene regulatory networks that contribute to complex diseases.
4. Genotype data and biological samples stored in databases and biobanks are available at any time when required, which can reduce waste caused by repeated experiments. Inbred mice and RIX lines can produce identical animals; the housing environment and experimental protocols are also the same, so the genotype data and standardized biological samples are worth storing.
5. National human disease repositories increase reliability through curation, preservation, genetic quality control, and protection from pathogens. To avoid inappropriate breeding, animal husbandry, and quality control, Repositories routinely analyze animals’ genomes before making them available and control for pathogens through frequent monitoring and the ability to revive the strain by IVF. The NAMR distributed over 2000 live engineered mice representing hundreds of mutant lines in 2022.

In recent years, tremendous efforts have been invested in identifying human genes or genomic regions involved in complex diseases and understanding their function. Moreover, human medicine is in the midst of a shift to “Personalized Medicine,” in which detailed information on patient pathology and DNA structure are gathered and combined to generate preventive and therapeutic solutions tailored to the patient’s needs. Human research has inherent limitations, including confounding environmental factors, population stratification, and the rarity of causative genetic variants. Many limitations in studies of human populations can be addressed in mice, which share human susceptibility to many pathogens and environmental circumstances. However, most mouse models only recapitulate part of the pathological features of human disease, and standard laboratory mouse lines have low genetic diversity and thus have limited usefulness for studying genetic variation in complex traits. Here, we aim to build a mice-version UK biobank. We strongly believe that the different mouse models, with their genotype data, phenotype data, and tissue samples, will offer a powerful platform for dissecting the complexity of human diseases.

## Data Availability

No dataset was generated or analyzed during this study.
